# A georeferenced dataset of nocturnal macrolepidoptera: A tool for forest management and biodiversity conservation

**DOI:** 10.1016/j.dib.2022.107882

**Published:** 2022-01-28

**Authors:** Stefano Scalercio, Carlo Di Marco, Nicola Puletti

**Affiliations:** a Council for Agricultural Research and Economics, Research Centre for Forestry and Wood, Via Settimio Severo 83, Rende I-87036, Italy; b Council for Agricultural Research and Economics, Research Centre for Forestry and Wood, Viale Santa Margherita 80, Arezzo I-52100, Italy

**Keywords:** Lepidoptera, Beech, Pine, Natura 2000 network, Sila National Park, Italy

## Abstract

In this paper we provide a georeferenced dataset of raw data concerning occurrence and abundance of nocturnal macrolepidoptera, an insect group largely recognized as a good ecological indicator of forest ecosystems. Data have been collected by using light traps located in 15 beech and 20 Calabrian black pine forest lots, 20 of which included in Natura 2000 sites. The sampling was carried out monthly lasting from May to late October 2019 and 2020 in order to cover the entire period during which favourable conditions for adult monitoring occurred, and to encompass phenological changes occurring across seasons in moth diversity. The dataset is composed by a total of 42,834 individuals belonging to 363 species. Due to the relatively small attractive radius of used light traps (about 25 m), georeferenced lepidopteran data can be easily correlated to any kind of spatial environmental variables and forest attributes and to their temporal variations being useful to quantify also the effects of long-term ecological drivers.

## Specifications Table

**Table:** 

Subject	Biodiversity
Specific subject area	Species richness and abundance of forest Lepidoptera communities
Type of data	Table
How the data were acquired	Data has been collected by using UV LED light traps [1].
Data format	Raw Filtered
Description of data collection	Data have been collected in 35 georeferenced sites located in pine and beech forests of the Sila National Park, Calabria region, South Italy (Table 1). Traps have been activated one night per month from the sunset to the sunrise under weather conditions favourable to the moth activity (Table 2). Specimens have been sorted, identified to species level, and counted in the laboratory (Supplementary material).
Data source location	Research Centre for Forestry and Wood Rende, Cosenza Italy
Data accessibility	Direct link to the dataset: https://data.mendeley.com/datasets/db7kwbxjyr/1

## Value of the Data

• Georeferenced datasets concerning biodiversity and abundance of insects in protected areas of the Mediterranean Basin are very rare and difficult to obtain mainly because of the specialisation needed to identify specimens to species level in hyper-diverse taxa.
• Entomologists, ecologists, and conservationists who investigate forest diversity and changes of forest ecosystems could be really interested in such datasets that can also be used by extrapolating data to study population dynamics and distribution of individual species.
• The georeferenced dataset we provided can be used in other studies devoted (i) to compare the community structure of insects of different geographic areas, (ii) to evaluate temporal changes of communities in the same sites as response, for example, to climate change, (iii) to carry out studies of landscape ecology. Entomologists can also use these data to (iiii) assess changes in the populations of defoliator species.

## 1 Data Description

This study includes abundance data of moths belonging to the so-called Macrolepidoptera, an insect group largely used as ecological indicator of forest ecosystems [2], [3], [4]. They were sampled in 35 georeferenced sites of the Sila National Park, South Italy (Table 1). Fifteen sites were in a beech forest and 20 in a Calabrian black pine forest. Two Habitat Directive sites were interested by sampling, namely the Special Areas of Conservation Pinete del Roncino (site code: IT9330117) and Colle del Telegrafo (site code: IT9330128). Sampling covered the territory of three municipality, all included in the Catanzaro Province, Italy, at an altitude comprised between 1170 and 1620 metres above the sea level (Table 1). Within sites we found a minimum of 36 and a maximum of 168 species, and a minimum of 389 and a maximum of 3360 individuals (Table 1).

**Table 1:** Characteristics of sampled sites and raw lepidopteran data.

Site	Forest type	Habitats Directive sites	Locality	Municipality	Altitude	Latitude - Longitude	Number of species	Number of individuals
code	dominant	code	toponym		(m)	Decimal degree	Raw lepidopteran data	
SL_Fa1	beech forest	none	Tempone Morello	Taverna	1595	39.1325°N - 16.5650°E	168	3360
SL_Fa2	beech forest	none	Tempone Morello	Taverna	1590	39.1276°N - 16.5674°E	164	1957
SL_Fa3	beech forest	none	Tempone Morello	Taverna	1580	39.1311°N - 16.5708°E	148	2269
SL_Fa4	beech forest	none	Tempone Morello	Taverna	1550	39.1291°N - 16.5727°E	139	1516
SL_Fa5	beech forest	none	Tempone Morello	Taverna	1580	39.1278°N - 16.5812°E	108	2405
SL_Fa6	beech forest	none	Colle del Telegrafo	Taverna	1580	39.1200°N - 16.5918°E	117	1581
SL_Fa7	beech forest	IT9330128	Colle del Telegrafo	Taverna	1570	39.1217°N - 16.5969°E	114	2611
SL_Fa8	beech forest	none	Colle del Telegrafo	Taverna	1590	39.1171°N - 16.5958°E	97	1201
SL_Fa9	beech forest	none	Colle del Telegrafo	Taverna	1620	39.1167°N - 16.6003°E	123	1890
SL_Fa10	beech forest	none	Colle del Telegrafo	Taverna	1615	39.1176°N - 16.6019°E	119	1853
SL_Fa11	beech forest	none	Colle del Telegrafo	Taverna	1610	39.1106°N - 16.6064°E	112	1940
SL_Fa12	beech forest	none	Tirivolo	Taverna	1580	39.1028°N - 16.6197°E	126	2648
SL_Fa13	beech forest	IT9330128	Capitano	Taverna	1560	39.0975°N - 16.6197°E	36	471
SL_Fa14	beech forest	none	Calistro	Zagarise	1575	39.0894°N - 16.6260°E	91	789
SL_Fa15	beech forest	none	Villaggio Buturo	Zagarise	1540	39.0766°N - 16.6353°E	99	806
SL_Ro1	pine forest	none	Fiume Simeri	Taverna	1208	39.0784°N - 16.5735°E	103	707
SL_Ro2	pine forest	IT9330117	Fiume Simeri	Taverna	1170	39.0841°N - 16.5749°E	75	397
SL_Ro3	pine forest	IT9330117	Cannapia	Albi	1213	39.0859°N - 16.5777°E	132	1078
SL_Ro4	pine forest	IT9330117	Cannapia	Albi	1223	39.0831°N - 16.5809°E	115	942
SL_Ro5	pine forest	IT9330117	Cannapia	Albi	1273	39.0815°N - 16.5850°E	90	534
SL_Ro6	pine forest	IT9330117	Cannapia	Albi	1247	39.0773°N - 16.5865°E	92	556
SL_Ro7	pine forest	IT9330117	Coturelle	Albi	1259	39.0734°N - 16.5891°E	133	1031
SL_Ro8	pine forest	IT9330117	Coturelle	Albi	1215	39.0707°N - 16.5910°E	144	1022
SL_Ro9	pine forest	IT9330117	Coturelle	Albi	1195	39.0671°N - 16.5919°E	125	1092
SL_Ro10	pine forest	IT9330117	Coturelle	Albi	1184	39.0658°N - 16.5967°E	88	389
SL_Ro11	pine forest	IT9330117	Roncino	Taverna	1270	39.0863°N - 16.5860°E	117	846
SL_Ro12	pine forest	IT9330117	Roncino	Taverna	1268	39.0910°N - 16.5867°E	114	913
SL_Ro13	pine forest	IT9330117	Roncino	Taverna	1275	39.0969°N - 16.5810°E	121	954
SL_Ro14	pine forest	IT9330117	Roncino	Taverna	1262	39.0947°N - 16.5902°E	113	670
SL_Ro15	pine forest	IT9330117	Roncino	Taverna	1235	39.0895°N - 16.5911°E	99	560
SL_Ro16	pine forest	IT9330117	Colle Roncino	Taverna	1363	39.0878°N - 16.5977°E	96	581
SL_Ro17	pine forest	IT9330117	Colle Roncino	Taverna	1375	39.0853°N - 16.5989°E	106	689
SL_Ro18	pine forest	IT9330117	Colle Roncino	Taverna	1432	39.0865°N - 16.6041°E	84	718
SL_Ro19	pine forest	IT9330117	Colle Roncino	Taverna	1454	39.0793°N - 16.6067°E	103	737
SL_Ro20	pine forest	none	Colle Roncino	Taverna	1449	39.0832°N - 16.6104°E	106	1121

**Fig. 1:**
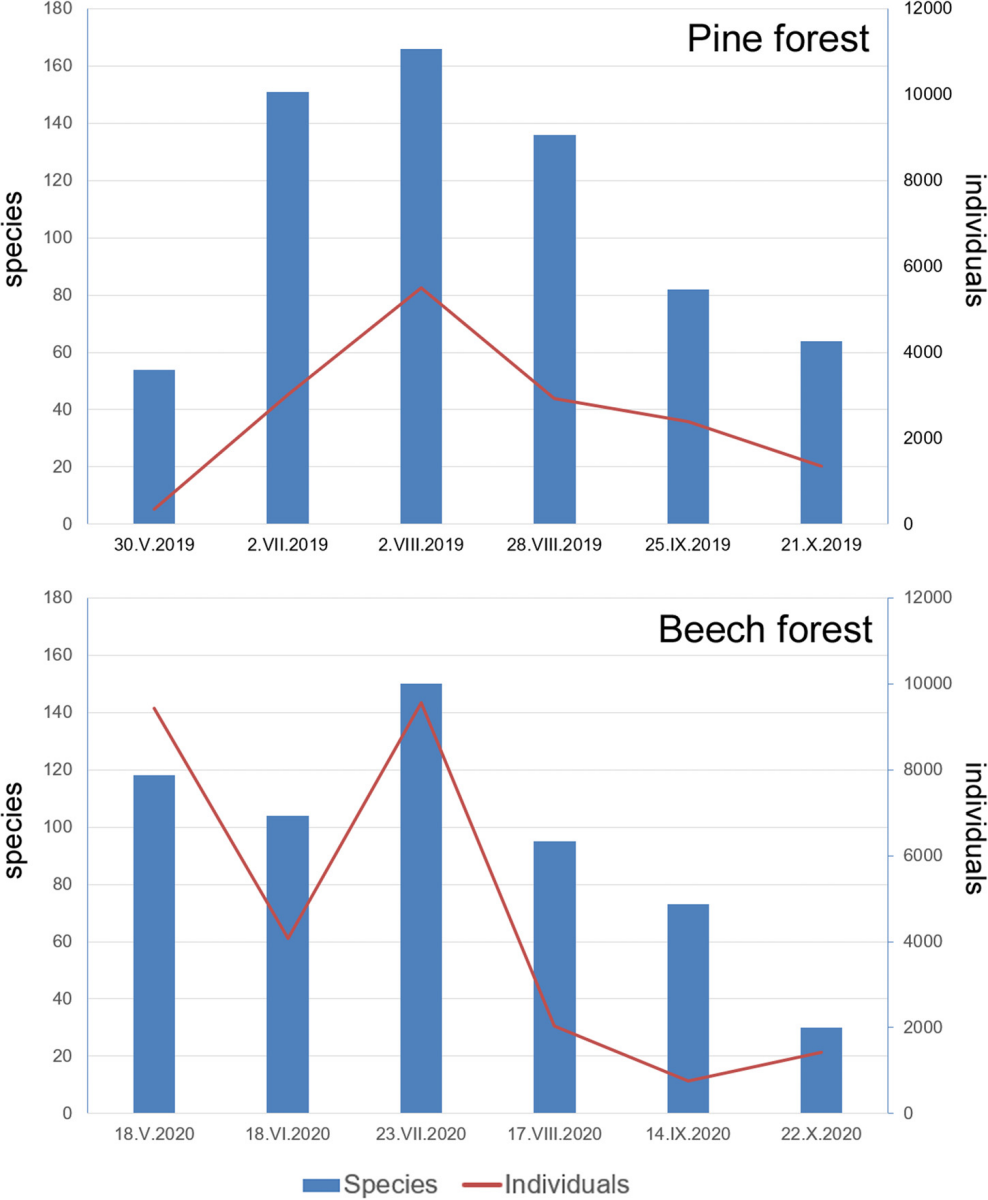
Distribution across time of species richness and abundance of individuals in sampled forest types.

Sites were sampled six times. Pine forests were sampled in 2019 and beech forests in 2020 (Table 2). We provided exact sampling nights in order to facilitate the recovering of weather conditions, moon stages, and other parameters that can affect moth captures, useful for future studies. For example, very favourable conditions occurred during the sampling of May 2020 in beech forests when we collected more species and individuals than in June (Fig. 1). In this study there is only data from one year for each type of forest and this fact can apparently limits the usefulness of this data for spatio-temporal ecological analysis. However, it has been demonstrated that beta-diversity is stable across years [5] allowing us to evaluate changes amongst communities also in years that experienced different weather conditions.

**Table 2:** Exact sampling nights for each site.

Site code	sampling nights					
SL_Fa1	2020-05-18	2020-06-18	2020-07-23	2020-08-17	2020-09-14	2020-10-22
SL_Fa2	2020-05-18	2020-06-18	2020-07-23	2020-08-17	2020-09-14	2020-10-22
SL_Fa3	2020-05-18	2020-06-18	2020-07-23	2020-08-17	2020-09-14	2020-10-22
SL_Fa4	2020-05-18	2020-06-18	2020-07-23	2020-08-17	2020-09-14	2020-10-22
SL_Fa5	2020-05-18	2020-06-18	2020-07-23	2020-08-17	2020-09-14	2020-10-22
SL_Fa6	2020-05-18	2020-06-18	2020-07-23	2020-08-17	2020-09-14	2020-10-22
SL_Fa7	2020-05-18	2020-06-18	2020-07-23	2020-08-17	2020-09-14	2020-10-22
SL_Fa8	2020-05-18	2020-06-18	2020-07-23	2020-08-17	2020-09-14	2020-10-22
SL_Fa9	2020-05-18	2020-06-18	2020-07-23	2020-08-17	2020-09-14	2020-10-22
SL_Fa10	2020-05-18	2020-06-18	2020-07-23	2020-08-17	2020-09-14	2020-10-22
SL_Fa11	2020-05-18	2020-06-18	2020-07-23	2020-08-17	2020-09-14	2020-10-22
SL_Fa12	2020-05-18	2020-06-18	2020-07-23	2020-08-17	2020-09-14	2020-10-22
SL_Fa13	2020-05-18	2020-06-18	2020-07-23	2020-08-17	2020-09-14	2020-10-22
SL_Fa14	2020-05-18	2020-06-18	2020-07-23	2020-08-17	2020-09-14	2020-10-22
SL_Fa15	2020-05-18	2020-06-18	2020-07-23	2020-08-17	2020-09-14	2020-10-22
SL_Ro1	2019-05-30	2019-07-02	2019-08-02	2019-08-28	2019-09-25	2019-10-21
SL_Ro2	2019-05-30	2019-07-01	2019-08-02	2019-08-28	2019-09-25	2019-10-21
SL_Ro3	2019-05-30	2019-07-02	2019-08-02	2019-08-28	2019-09-25	2019-10-21
SL_Ro4	2019-05-30	2019-07-02	2019-08-02	2019-08-28	2019-09-25	2019-10-21
SL_Ro5	2019-05-30	2019-07-02	2019-08-02	2019-08-28	2019-09-25	2019-10-21
SL_Ro6	2019-05-30	2019-07-01	2019-08-02	2019-08-28	2019-09-25	2019-10-21
SL_Ro7	2019-05-30	2019-07-01	2019-08-02	2019-08-28	2019-09-25	2019-10-21
SL_Ro8	2019-05-30	2019-07-01	2019-08-02	2019-08-28	2019-09-25	2019-10-21
SL_Ro9	2019-05-29	2019-07-01	2019-08-02	2019-08-28	2019-09-25	2019-10-21
SL_Ro10	2019-05-29	2019-07-01	2019-08-02	2019-08-28	2019-09-25	2019-10-21
SL_Ro11	2019-05-29	2019-07-01	2019-08-02	2019-08-28	2019-09-25	2019-10-21
SL_Ro12	2019-05-29	2019-07-01	2019-08-02	2019-08-28	2019-09-25	2019-10-21
SL_Ro13	2019-05-29	2019-07-01	2019-08-02	2019-08-28	2019-09-25	2019-10-21
SL_Ro14	2019-05-29	2019-07-01	2019-08-02	2019-08-28	2019-09-25	2019-10-21
SL_Ro15	2019-05-29	2019-07-01	2019-08-02	2019-08-28	2019-09-25	2019-10-21
SL_Ro16	2019-05-29	2019-07-01	2019-08-02	2019-08-28	2019-09-25	2019-10-21
SL_Ro17	2019-05-29	2019-07-01	2019-08-02	2019-08-28	2019-09-25	2019-10-21
SL_Ro18	2019-05-29	2019-07-01	2019-08-02	2019-08-28	2019-09-25	2019-10-21
SL_Ro19	2019-05-29	2019-07-01	2019-08-02	2019-08-28	2019-09-25	2019-10-21
SL_Ro20	2019-05-29	2019-07-01	2019-08-02	2019-08-28	2019-09-25	2019-10-21

The complete list of species, their abundance as number of individuals, and presence in sampled forest sites is reported in Supplementary material. Species are grouped by Family and listed in alphabetical order. Nomenclature follows Karsholt and van Nieukerken Lepidoptera [6] with exceptions for few recently recognized species (*Hylaea mediterranea* [7], *Nothocasis rosariae* [8], *Tephronia theophilaria* [9], *Hoplodrina alsinides* [10]). The total number of individuals and the total number of sites where a species has been collected are also reported (Supplementary material).

## 2 Experimental Design, Materials and Methods

Sampling sites have been chosen in order to be (i) representative of vegetal cover and structure of investigated forest types, (ii) easy to reach by operators but far enough from roads to minimise the effects on moth communities, and (iii) not visible from passing cars.

Sites were georeferenced and traps have been settled in the same points six nights per year from May to October with about 4 weeks of interval. Sampling nights have been chosen during weather conditions favourable to moth activity, i.e. temperature near or higher than the mean of the period, no or low wind, no or light rain, one week before or after the new moon occurrence [3].

Moths have been collected using light traps equipped with UV LEDs (315–400 nm, light angle 120°) as those illustrated in Infusino et al. [1], powered by a 15 A and 12 V battery, and with ethyl acetate as killing agent.

Traps worked simultaneously in each forest type, with very few exceptions due to technical problems (Table 1). Light traps were settled and turned on before dusk, then unsettled the morning after. Collected specimens were put in small jars with blotting paper and few drops of ethyl acetate and taken to the Wildlife management and forest biodiversity laboratory of the Research Centre of Forestry and Wood, Rende, Italy. Only specimens within traps have been considered.

Sorting, identification of species and counting of individuals have been carried out in the laboratory. Identification has been carried out by comparing specimens with those in the research collection of the laboratory and with available iconography concerning European moth fauna. Most difficult species needed extraction of genitalia for correct identification following the protocol in Berio [11]. Voucher specimens have been stored in the laboratory collection of Lepidoptera.

## Ethics Statements

The authors declare that the present work did not include experiments on human subjects and/or animals.

## CRediT authorship contribution statement

**Stefano Scalercio:** Conceptualization, Methodology, Investigation, Data curation, Writing – original draft, Supervision. **Carlo Di Marco:** Methodology, Investigation. **Nicola Puletti:** Conceptualization, Methodology, Funding acquisition.

## Declaration of Competing Interest

The authors declare that they have no known competing financial interests or personal relationships that could have appeared to influence the work reported in this paper.

## Appendix Supplementary materials

Image, application 1
